# From Answers to Insights: Unveiling the Strengths and Limitations of ChatGPT and Biomedical Knowledge Graphs

**DOI:** 10.21203/rs.3.rs-3185632/v1

**Published:** 2023-08-01

**Authors:** Yu Hou, Jeremy Yeung, Hua Xu, Chang Su, Fei Wang, Rui Zhang

**Affiliations:** University of Minnesota; University of Minnesota; Yale University; Temple University; Weill Cornell Medicine; University of Minnesota

**Keywords:** Large Language Models (LLMs), ChatGPT, Biomedical Knowledge Graph (BKG)

## Abstract

**Purpose:**

Large Language Models (LLMs) have shown exceptional performance in various natural language processing tasks, benefiting from their language generation capabilities and ability to acquire knowledge from unstructured text. However, in the biomedical domain, LLMs face limitations that lead to inaccurate and inconsistent answers. Knowledge Graphs (KGs) have emerged as valuable resources for organizing structured information. Biomedical Knowledge Graphs (BKGs) have gained significant attention for managing diverse and large-scale biomedical knowledge. The objective of this study is to assess and compare the capabilities of ChatGPT and existing BKGs in question-answering, biomedical knowledge discovery, and reasoning tasks within the biomedical domain.

**Methods:**

We conducted a series of experiments to assess the performance of ChatGPT and the BKGs in various aspects of querying existing biomedical knowledge, knowledge discovery, and knowledge reasoning. Firstly, we tasked ChatGPT with answering questions sourced from the “Alternative Medicine” sub-category of Yahoo! Answers and recorded the responses. Additionally, we queried BKG to retrieve the relevant knowledge records corresponding to the questions and assessed them manually. In another experiment, we formulated a prediction scenario to assess ChatGPT’s ability to suggest potential drug/dietary supplement repurposing candidates. Simultaneously, we utilized BKG to perform link prediction for the same task. The outcomes of ChatGPT and BKG were compared and analyzed. Furthermore, we evaluated ChatGPT and BKG’s capabilities in establishing associations between pairs of proposed entities. This evaluation aimed to assess their reasoning abilities and the extent to which they can infer connections within the knowledge domain.

**Results:**

The results indicate that ChatGPT with GPT-4.0 outperforms both GPT-3.5 and BKGs in providing existing information. However, BKGs demonstrate higher reliability in terms of information accuracy. ChatGPT exhibits limitations in performing novel discoveries and reasoning, particularly in establishing structured links between entities compared to BKGs.

**Conclusions:**

To address the limitations observed, future research should focus on integrating LLMs and BKGs to leverage the strengths of both approaches. Such integration would optimize task performance and mitigate potential risks, leading to advancements in knowledge within the biomedical field and contributing to the overall well-being of individuals.

## 1 Introduction

Recently the Large Language Models (LLMs) have exhibited exceptional performance across a diverse range of natural language processing tasks^1–3^. LLMs, especially the GPT-3.5 and GPT-4, are powerful models trained on vast amounts of textual data, enabling them to generate human-like text and perform various language-related tasks^4^. These models have shown great performances in diverse domains, including chatbots, question-answering systems, and language translation, among others. Their ability to understand and generate text has sparked interest in exploring their potential to replace traditional knowledge resources.

Knowledge Graphs (KGs) serve as valuable repositories of structured information and have gained significant attention due to their ability to represent and organize knowledge in a structured manner. They facilitate knowledge discovery, entity linking, and semantic querying, making them essential for various applications, including information retrieval, recommendation systems, and semantic search. In recent years, the field of biomedicine has witnessed the emergence of Biomedical Knowledge Graphs (BKGs) as a novel paradigm for managing large-scale and heterogeneous biomedical knowledge, which have garnered considerable interest in the biomedical community^5–10^. A BKG is a multi-relational graph or network that integrates, harmonizes, and stores biomedical knowledge acquired from single or multiple expert-derived knowledge sources. Over the past decade, substantial efforts have been dedicated to constructing BKGs by integrating diverse expert-curated knowledge bases^6,8,11–13^ and extracting knowledge from literature using natural language processing (NLP) techniques^14–16^. Consequently, numerous distinct BKGs have been developed^17–20^.

LLMs exhibit impressive language generation capabilities and have the potential to acquire knowledge from vast amounts of unstructured text. They can generate responses to questions and provide valuable insights. However, LLMs face several limitations when confronted with the biomedical domain, leading to issues like erroneous and inconsistent answers^21–23^. This study aims to evaluate and compare the ChatGPT (a popular LLM) and BKG through comprehensive assessments encompassing querying existing biomedical knowledge, discovering novel knowledge, and providing reasoning capabilities. We shed light on the strengths and limitations of ChatGPT and existing KG, providing insights into their complementary roles in knowledge representation and utilization. Our findings contribute to the ongoing discussions surrounding the synergies and potential collaborations between LLMs and KGs in enhancing knowledge-driven applications.

## 2 Methods

To evaluate the effectiveness of ChatGPT and BKGs in biomedicine, we conducted a comprehensive comparative analysis. Specifically, we first assessed their performance in answering drug-related and dietary supplements (DS)-related question answering. Next, we evaluated their capacities in novel biomedical knowledge discovery, e.g., drug and DS repurposing. Last, we also assessed the comprehensiveness of the biomedical knowledge they provided. Specifically, we investigated ChatGPT’s ability to generate accurate and relevant responses to drug-related and DS-related queries and its potential for knowledge discovery by identifying hidden patterns and relationships.

### 2.1 Compared Methods

The integrated Dietary Supplements Knowledge Base (iDISK)^24^, which serves as an encompassing knowledge graph comprising a diverse range of dietary supplements, including vitamins, herbs, minerals, and other relevant entities. iDISK has been meticulously standardized and integrated from multiple widely used and authoritative dietary supplement resources, namely the Natural Medicines Comprehensive Database (NMCD)^25^, Memorial Sloan Kettering Cancer Center (MSKCC)^26^, the Dietary Supplement Label Database (DSLD)^27^, and the Natural Health Products Database (NHP), comprised of the Natural Health Product Ingredients Database^28^ and the Licensed Natural Health Products Database^29^. This integrated knowledge base incorporates various attributes and relationships that provide comprehensive information about each dietary supplement, including details such as its inclusion as an ingredient in specific products and its potential interactions with medications. In this study, iDISK will serve as the primary BKG for investigating and analyzing DS-related exploration tasks.

The integrative Biomedical Knowledge Hub (iBKH)^30^ was developed through a meticulous process of harmonizing and integrating information from a diverse range of biomedical resources. This comprehensive knowledge hub incorporates data from 18 highly regarded and carefully curated sources. The current iteration of iBKH encompasses a vast collection of over 2.2 million entities, representing 11 distinct entity types. Furthermore, it encompasses 45 different types of relations that establish connections between various entity pairs, spanning across 18 different categories. In the context of this study, iBKH will serve as the primary Biomedical Knowledge Graph (BKG) for conducting thorough investigations and analyses of drug-related exploration tasks.

ChatGPT^31^, developed by OpenAI, is an advanced conversational AI model that utilizes the GPT (Generative Pre-trained Transformer) architecture^4^. It is designed to generate human-like responses to text-based inputs and has garnered significant attention for its language generation capabilities and natural language processing performance. ChatGPT 3.5 and 4.0 are the latest versions of the model. The main differences between ChatGPT 3.5 and 4.0 are in the following aspects: Model scale: GPT-4 is larger than GPT-3.5, containing more parameters and computational power, which allows it to handle more complex tasks and language patterns. ChatGPT 4 has significantly progressed over ChatGPT 3.5, offering better language understanding, enhanced conversational abilities, and broader potential applications. In this study, we aim to evaluate the performance of ChatGPT, specifically the GPT-3.5 and GPT-4.0 versions, in the context of question-answering, biomedical knowledge discovery, and reasoning tasks within the biomedical domain.

### 2.2 Performance Evaluation of ChatGPT and BKG in Question-Answering

We used the question-answering (Q&A) dataset (including their titles and contents) from the “Alternative Medicine” sub-category in Yahoo! Answers^32^. The questions were grouped into categories such as Adverse Effects, Background, Contraindication, Effectiveness, Indication, Interaction, Safety, Uncertain, Unclassified, and Usage. Initially, we randomly selected 5 questions from each group, resulting in a total of 50 questions.

#### Q&A based on ChatGPT.

To collect responses from ChatGPT, we input the questions as prompts and record the generated answers.

#### Q&A based on BKGs.

**Figure 1:**
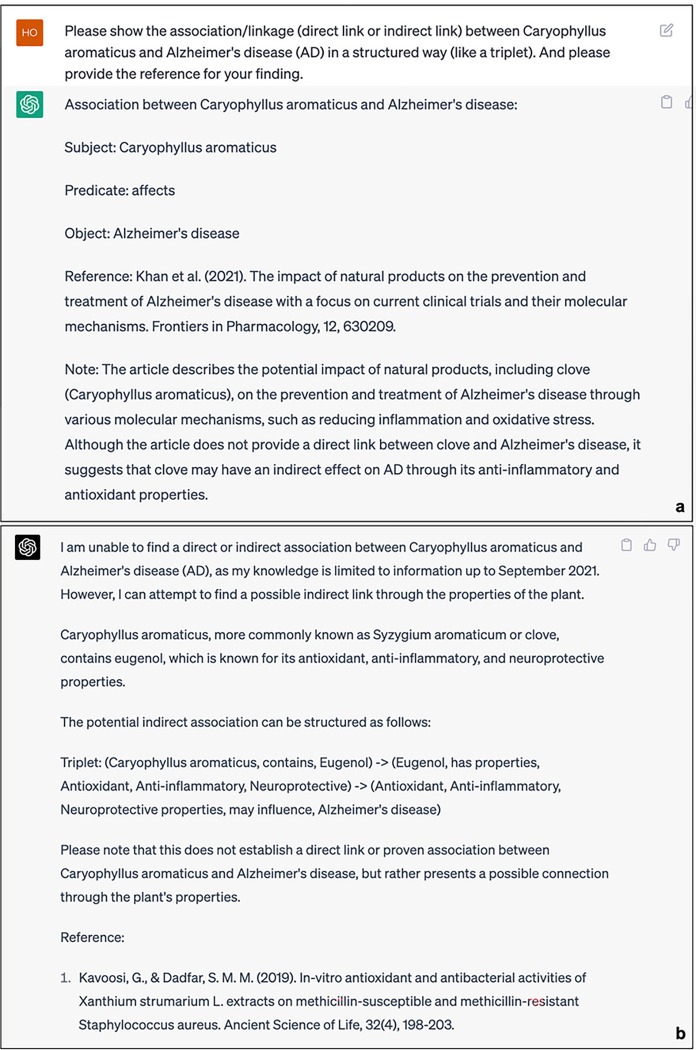
Responses of ChatGPT to the Knowledge Reasoning task (DS). a. Results from GPT-4.5. b. Result from GPT-4. Abbreviation: DS = Dietary Supplements

The dataset utilized for evaluating ChatGPT’s query performance on existing biomedical knowledge was sourced from the “Alternative Medicine” sub-category of Yahoo! Answers^32^. Consequently, iDISK^24^ will be employed to explore the pertinent answers to the given questions. Initially, we identified the unique identifier of the subject and its corresponding relationship based on the question description. Subsequently, we established connections between the object identifiers and the identifiers of relevant supplements, ingredients, therapeutic effects, and/or adverse effects using the iDISK Relationship Table, depending on the specific question at hand. Lastly, we retrieved the names of the relevant concepts and transformed the findings into natural language to provide a comprehensive response to the original query. For instance, to address the question “What are the side effects for panax ginseng?”, we first located the concept ID of panax ginseng within iDISK and proceeded to identify the corresponding relationship mentioned in the question, which in this case is “has_adverse_reaction,” within the relationship table. Subsequently, we retrieved the entities associated with panax ginseng and the relation “has_adverse_reaction,” and translated these records into natural language to formulate the final answer. A visual representation of the overall query process is depicted in Fig. 1.

#### Q&A performance evaluation.

To evaluate the responses, we followed the LiveQA Track guidelines^33^ and assigned judgment scores on a scale ranging from 0 to 3. Two experts who have medical backgrounds were introduced for manual scoring. A score of 0 indicates an incorrect response (poor or unreadable response), 1 indicates an incorrect but related answer (fair response), 2 denotes a correct but incomplete response (good response), and 3 indicates a correct and complete answer (excellent response). Based on this scale, we calculated two metrics. Firstly, we computed the average score, which evaluated the first retrieved answer for each test question^33,34^. Secondly, we measured the *succ*@*i*+ metric, which is defined as the ratio of the number of questions with a score ≥ *i*(we considered *i* rangng from 1 to 3) to the total number of questions. For example, *succ*@1+means the percentage of questions that were answered by the conversational agent (CA) with at least a fair grade^33^. To assess the statistical differences in the performance of the three systems (ChatGPT 4.0, ChatGPT 3.5 and iDISK), we used the t-test for normal distributed data or Mann-Whitney U test for non-normal distributed data. The QQ-plot was performed to look at the normality of the data. The analysis is conducted using R 1.1 with the package “car^35^”.

### 2.3 Performance Evaluation of ChatGPT and BKG in Knowledge Discovery

To test knowledge discovery capabilities between ChatGPT and BKGs, we devised a prediction scenario that emulates the task of drug and DS repurposing for Alzheimer’s Disease (AD).

#### AD drug/DS repurposing based on ChatGPT.

The task was to prompt ChatGPT to suggest drugs or DSs that are not presently utilized for the treatment or prevention of AD but possess the potential to be employed in such capacities. Each prompt was repeated 10 times, and we collected all the results returned by ChatGPT. The specifically crafted prompts included:

1. Please provide the approved drugs that are not currently used to treat Alzheimer’s disease but are potentially available for the treatment of AD. And please give your rationale. (Drug)
2. Please provide which dietary supplements have the potential to treat/prevent Alzheimer’s disease. And please give your rationale. (DS)

We examined the answers generated by ChatGPT to determine if these answers met the following criteria:

1. whether they were already present in existing BKGs (specifically, iBKH^30^ for drugs and ADInt^36^ for DSs);
2. whether they were documented in clinical trials; and 3. whether they were supported by existing literature.

#### AD drug/DS repurposing based on BKG.

Building upon our previous research^30,36^, we employed knowledge graph embedding (KGE) algorithms to compute machine-readable embedding vectors for entities and relations within the BKGs (iBKH and ADInt) while preserving the graph structure. Subsequently, we leveraged these learned embedding vectors to conduct link prediction, enabling the prediction of potential relations between pairs of entities. Then, we generated suggested potential drug and DS candidates for AD. This approach involved identifying relationships that were absent in the existing BKGs, thus enabling the exploration of novel therapeutic possibilities in the context of AD.

### 2.4 Performance Evaluation of ChatGPT and BKG in Knowledge Reasoning

To assess the comprehensiveness of ChatGPT’s knowledge base, we further examined its capability in establishing associations between the proposed drug and DS candidates with AD. In our previous study, we investigated potential pharmaceuticals and DS for the treatment or prevention of AD using link prediction techniques^30,36^. Building upon these previous findings, our objective was to evaluate ChatGPT’s knowledge base by examining the associations it provides between these hypothetical drug/DS candidates and AD, as well as the corresponding references it offers to support these hypotheses. To accomplish this, we formulated scenario-based inquiries as follows:

1. Please show the association/linkage (direct link or indirect link) between [Tested Drug] and Alzheimer’s disease (AD) in a structured way (like a triplet). And please provide the reference for your finding.
2. Please show the association/linkage (direct link or indirect link) between [Tested DS] and Alzheimer’s disease (AD) in a structured way (like a triplet). And please provide the reference for your finding.

## 3 Results

We conducted a comprehensive evaluation of the performance of three systems, namely GPT-3.5, GPT-4.0 and iDISK, in addressing a set of 43 questions. Table 1 demonstrated the performance of three systems in the Q&A task. First, even the early version of ChatGPT (GPT-3.5) can achieve a comparable performance in biomedical Q&A of the BKG (iDSIK)-based approach (p-value: 0.20). Notably, the latest version of ChatGPT (GPT-4.0) demonstrated a better performance than iDSIK and ChatGPT (GPT-3.5) (both p-value < 0.05) with an average score of 2.12 ± 0.83, outperforming iDISK (average score: 1.64 ± 0.81) and GPT-3.5 (average score: 1.44 ± 0.98). In terms of providing references, iDISK surpassed ChatGPT (both GPT-4.0 and GPT-3.5) by offering the name of the database from which the data was retrieved. Conversely, ChatGPT (both GPT-4.0 and GPT-3.5) fell short in providing valid references, as the mentioned article names and/or authors were found to be fabricated.

Table 2 presents the results obtained from the conversation questions using ChatGPT (model GPT-3.5) to simulate a scenario related to drug/DS repositioning. Upon conducting ten iterations of the question, we observed that ChatGPT (GPT-3.5) returned several identical responses. Specifically, Levetiracetam was suggested in 6 out of the ten iterations, while Lithium and Ibuprofen were each suggested in 5 iterations. Moreover, the majority of drugs suggested by ChatGPT (GPT-3.5) have been investigated for potential associations with Alzheimer’s Disease treatment in ClinicalTrials.gov. And all the drugs have been investigated in one or more scientific literature that explores their potential association with Alzheimer’s Disease. Upon examining these drugs within a comprehensive biomedical knowledge graph (iBKH), it was observed that 12 out of 16 drugs suggested by ChatGPT (GPT-3.5) are present in iBKH with direct links to AD. For those drugs lacking direct links, the shortest distance between them and Alzheimer’s Disease is only 2, with reasonable paths existing, such as (Drug) - [Target_DG] - (Gene) - [Associate_DiG] - (AD). Analogous results are observed in the employment of ChatGPT (GPT-3.5) to simulate exploratory scenarios involving novel DS that may potentially treat or prevent AD. In addition, all of the DSs recommended by ChatGPT (GPT-3.5) have been included in AD clinical trials. These DSs have been extensively researched for their correlation with AD, with findings documented in relevant academic publications. Furthermore, direct connections between the suggested DSs and AD can be identified within ADInt, a comprehensive knowledge graph encompassing AD-related concepts and various potential interventions. The recorded pathway in ADInt is denoted as (DS) - [Treats/Prevents] - (AD). Additionally, we executed a parallel experimental manipulation employing ChatGPT (GPT-4.0). Despite the enhanced diversity of the recommended drugs, they remained present in established clinical trials and literature. Concurrently, the associated pathway records persisted within the extant knowledge graph.

We employed the KGE model to obtain the embedding vectors of the Biomedical Knowledge Graphs (BKGs). Subsequently, link prediction was performed based on the embedding information, enabling the generation of potential drug and DS candidates for the treatment or prevention of AD. Importantly, these candidates have not been approved or involved in clinical trials for AD treatment. For instance, Loperamide, commonly used to treat diarrhea and frequently employed in inflammatory bowel disease, has shown potential implications in AD pathology. Research has indicated that Loperamide targets opioid receptors^37,38^, which have been suggested to be potentially linked to AD pathology^39^. Furthermore, Choerospondias axillaris, also known as Nepali hog plum, is a fruit with sour flesh and yellow skin. In a study^40^, it was discovered that Choerospondias axillaris inhibits both TNF protein and interleukin-6. These two inflammation mediators are well-known inducers of AD, as demonstrated in previous studies^41,42^.

**Figure 2:**
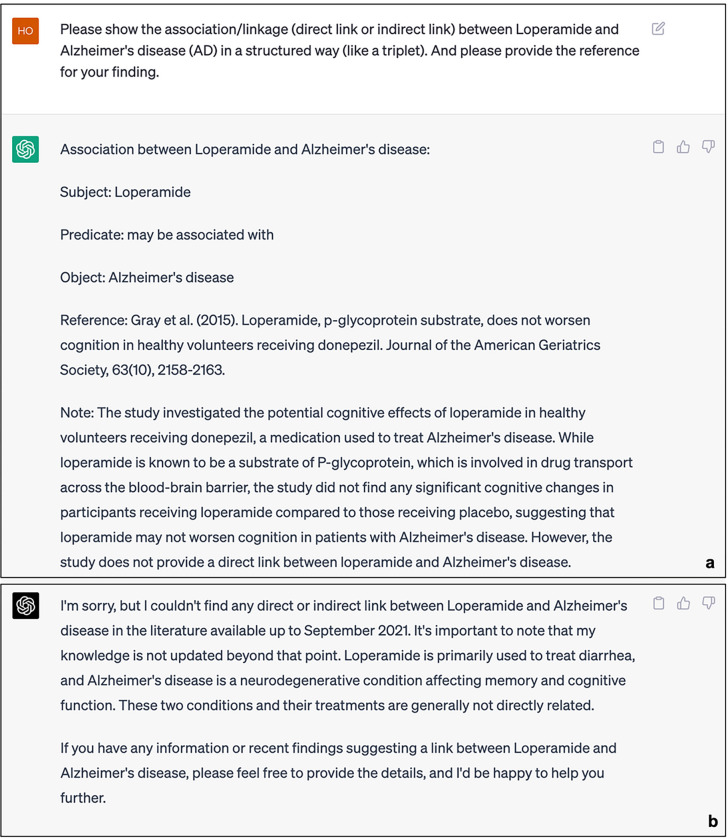
Responses of ChatGPT to the Knowledge Reasoning task (Drug). a. Results from GPT-3.5. b. Results from GPT-4.0.

Figure 2 presents the responses of ChatGPT (GPT-3.5) and ChatGPT (GPT-4.0) to the devised scenario query 1, respectively. An examination of their replies reveals that ChatGPT endeavored to establish a structured connection between the hypothetical DS (Caryophyllus aromaticus) and Alzheimer’s disease (AD). Nonetheless, upon verification of the references provided by ChatGPT, it becomes evident that these citations are not authentic. Similar outcomes were observed during the investigation of novel drugs in the designed scenario 2. Figure 3 illustrates the attempts made by ChatGPT (GPT-3.5) and ChatGPT (GPT-4.0) to present structured links between the hypothetical drug (Loperamide) and AD. The connection offered by GPT-3.5 continues to be unsuccessful in validating the authenticity of the provided reference. Moreover, the response generated by GPT-4.0 indicates that it could not establish any potential direct or indirect association between Loperamide and AD. In the BKG, we can readily identify the connection between Caryophyllus aromaticus and Alzheimer’s Disease (AD) through a specific pathway. The shortest path from Caryophyllus aromaticus to AD in ADInt is represented as (Caryophyllus aromaticus) - [INHIBITS] - (Kynurenine) - [AFFECTS] - (AD). Importantly, there is available literature that provides evidence supporting this association^43,44^. In addition, the shortest path from Loperamide to AD in iBKH is represented as (Loperamide) – [TARGET] – (Opioid Receptors) – [ASSOCIATE_WITH] – (AD), and the existing literature could support the pathway^37–39^.

## 4 Discussion

During the process of querying existing information, both ChatGPT (GPT-3.5) and BKG exhibited comparable capabilities, demonstrating their effectiveness in providing relevant information based on user queries. However, with the introduction of ChatGPT (GPT-4.0), a notable improvement in performance was observed compared to GPT-3 and BKG. The enhanced capabilities of ChatGPT (GPT-4.0) allowed for more accurate and comprehensive responses, surpassing the performance of both GPT-3 and KGs in this context. However, when it comes to the reliability of information sources, KG exhibited a clear advantage. KGs are built upon curated and structured knowledge from trusted sources, ensuring the reliability and accuracy of the information contained within them. This advantage stems from the rigorous data collection and validation processes employed in constructing BKG. On the other hand, ChatGPT’s responses are generated based on patterns and associations learned from a vast amount of text data, which may include both reliable and unreliable sources. As a result, while ChatGPT can provide quick responses, there may be a higher risk of encountering misinformation or inaccuracies compared to BKG. Therefore, when considering the reliability and trustworthiness of the information provided, KGs offer a more dependable and robust solution. Consequently, our findings highlight the potential benefits of integrating knowledge graph-based approaches with ChatGPT to enhance its domain-specific knowledge and overall performance in specialized applications. Further research is required to explore the feasibility of this integration and its implications on the efficacy of ChatGPT in diverse domains.

In addition, we discovered that ChatGPT is unable to perform the novel finding task based on existing knowledge, which is a critical limitation when considering its application in scientific research and discovery. In the second experiment, we attempted to simulate drug repurposing using ChatGPT as a means to generate innovative insights. The results of this experiment revealed that ChatGPT primarily provided outputs that were derived from pre-existing information. This information could either be directly queried within a knowledge graph or easily found in relevant resources, suggesting that ChatGPT’s capacity for generating truly novel findings is limited. These outcomes can be attributed to the underlying training data and architecture of ChatGPT, which is designed to draw upon its vast knowledge base to produce contextually relevant and coherent responses, rather than extrapolate new insights or hypothesize potential connections. This limitation highlights the need for developing advanced AI models that can not only process and comprehend existing knowledge but also deduce novel findings by identifying hidden patterns and relationships.

Our investigation has uncovered limitations in the knowledge reasoning capabilities of ChatGPT. Specifically, it lacks the ability to establish structured links between two entities based on existing knowledge. In our third experiment, we assessed the performance of ChatGPT in comparison to the BKG with respect to the establishment of relationships between entities. The results demonstrated that ChatGPT was unable to provide a structured link between two entities as effectively as the knowledge graph, underscoring its shortcomings in this specific task. Furthermore, the credibility of the results returned by ChatGPT emerged as a significant concern. Our findings indicated that the accuracy of its responses necessitates further verification, as the information provided by ChatGPT may not always be reliable. In the third experiment, it became evident that none of the references furnished by ChatGPT were genuine, casting doubt on the trustworthiness of the information it generated. These observations highlight the need for rigorous validation and verification mechanisms when employing ChatGPT for tasks that require high levels of accuracy and credibility. Consequently, future research should explore strategies to enhance the reliability of ChatGPT’s outputs, such as incorporating external validation sources or refining its training data to improve its capacity to provide accurate and credible information.

## 5 Conclusion

Our study assesses the capabilities of ChatGPT and existing BKGs in question answering, knowledge discovery, and knowledge reasoning. While ChatGPT with GPT-4.0 outperformed both GPT-3.5 and BKGs (both are comparable) to provide existing information; BKGs exhibited a clear advantage in terms of information reliability over both GPT models. Our findings revealed limitations in ChatGPT’s ability to perform novel discoveries based on existing knowledge. Furthermore, our investigation highlighted ChatGPT’s limitations in providing reasoning for knowledge discovery (e.g., establishing structured links between entities compared to BKGs). In conclusion, future investigations should prioritize the development of methodologies that integrate LLMs and BKGs, allowing researchers to harness the unique capabilities of each approach. This holistic approach would not only optimize task performance but also enable mitigating potential risks, thus advancing knowledge in the biomedical field and contributing to the overall well-being of individuals.

## Figures and Tables

**Table 1.** Performance of three systems in the Q&A task

Measurement	Systems		
	ChatGPT (GPT-4.0)	ChatGPT (GPT-3.5)	iDISK
Average Score	2.12 ± 0.83	1.44 ± 0.98	1.64 ± 0.81
succ@1 +	1.0	0.79	0.88
succ@2+	0.70	0.51	0.61
succ@3+	0.42	0.14	0.12

For succ@*i*+ metric, a higher value indicates the better performance.

**Table 2.** Results of simulating Drug/DS repositioning using ChatGPT.

Name	Type	Suggested count in ten iterations	In Clinical Trail	In literature	In BKGs	
					Direct link	The length of shortest Path
Levetiracetam	Drug	6	Yes	Yes^45,46^	No	2
Lithium	Drug	5	Yes	Yes^47^	Yes	1
Ibuprofen	Drug	5	Yes	Yes^48,49^	Yes	1
Rapamycin	Drug	1	Yes	Yes^50^	Yes	1
Pioglitazone	Drug	2	Yes	Yes^51^	Yes	1
Nilotinib	Drug	2	Yes	Yes^52^	No	2
Nitroglycerin	Drug	2	No	Yes^53^	Yes	1
Valproic acid	Drug	2	Yes	Yes^54,55^	Yes	1
Prazosin	Drug	2	Yes	Yes^56^	Yes	1
Insulin	Drug	1	Yes	Yes^57^	No	2
Cannabinoids	Drug	1	Yes	Yes^58–59^	Yes	1
Trazodone	Drug	1	Yes	Yes^60^	Yes	1
Riluzole	Drug	1	Yes	Yes^61–62^	Yes	1
Methylene blue	Drug	1	Yes	Yes^63^	Yes	1
Niacin	Drug	1	Yes	Yes^64^	Yes	1
Acamprosate	Drug	1	Yes	Yes^65^	No	2
Omega-3 fatty acids	DS	10	Yes	Yes^66^	Yes	1
Vitamin E	DS	10	Yes	Yes^67^	Yes	1
Curcumin	DS	9	Yes	Yes^68^	Yes	1
Ginkgo biloba	DS	6	Yes	Yes^69^	Yes	1
Vitamin B12	DS	3	Yes	Yes^70^	Yes	1
Vitamin D	DS	3	Yes	Yes^71^	Yes	1
Resveratrol	DS	1	Yes	Yes^72^	Yes	1
Vitamin B6	DS	1	Yes	Yes^73^	Yes	1

DS: Dietary Supplements

We used iBKH to query drug-related results and ADInt to query DS-related results.

## Data Availability

iBKH data is publicly available online at https://github.com/wcm-wanglab/iBKH. And ADInt data is available in the following Google Drive: https://drive.google.com/drive/folders/187HnI2d-RRFeYk_C7MYSCHtVX-6IuNVS?usp=sharing.
